# The Everyday Life of Patients With Tuberculosis in the Concentration Camp of Mittelbau-Dora (1943–1945)

**DOI:** 10.3389/fmed.2020.526839

**Published:** 2020-09-25

**Authors:** Philipp Kiosze, Florian Steger

**Affiliations:** Institute of the History, Philosophy and Ethics of Medicine, Ulm University, Ulm, Germany

**Keywords:** tuberculosis, national socialism, lung diseases, concentration camps, prisoners, medical records, World War II, medical staff

## Abstract

The everyday life of patients with tuberculosis in the main prisoner infirmary of the Mittelbau-Dora concentration camp is analyzed historically-critically by medical records, documents of the Schutzstaffel (SS) physicians, contemporary medical textbooks and memoirs of former inmates from partly international archives. To compare the medical treatment in the three phases of the concentration camp, the representative months of February 1944, July 1944 and January 1945 were examined. The analysis shows that SS hygienists inspected the place for fear of a collapse of the V-2 rocket production. The primitive medical infrastructure was slowly expanded after its founding in 1943. SS physicians and medics led and supervised the treatment provided by inmates. These were in an ethical dilemma between cooperation with the SS and commitment to the sick prisoners. The Tuberculosis Department was used for isolation. Sputum diagnostics and X-ray equipment were utilized as selection tools. Infectious patients laid usually for weeks in the same bed with two other patients. Significantly more resources were available, however, for non-infectious tuberculosis patients. The therapy was based on the medical expert opinion of the time and was mainly symptomatic such as fever reduction. Rest and vitamins should make prisoners fit for the armament industry. Patients with tuberculosis had a high death rate. The prisoners who survived were discharged, but often did not recover. Several thousand prisoners were selected for transports, which led to special concentration camps for seriously ill prisoners (Lublin-Majdanek, Bergen-Belsen) and the subcamp Boelcke-Kaserne. There, they often died of catastrophic conditions or were killed.

## Introduction

The Mittelbau-Dora concentration camp near Nordhausen in northern Thuringia was established on 28 August 1943. Initially it was founded as a subcamp of the Buchenwald concentration camp to construct the underground production of the V-2 rocket developed by Wernher von Braun (1912–1977). In the fall of 1944, it became an independent main camp, forming a concentration camp complex of 39 subcamps until early 1945. On 11 April 1945 it was liberated by the U.S. Army. Out of 60,000 almost exclusively male prisoners interned in this camp, 20,000 died as a result of catastrophic conditions. The Mittelbau-Dora concentration camp exemplifies the economic transformation of the concentration camps at the end of the Second World War. There, inmates were exploited under terrible conditions for the construction of underground tunnel systems for the armament industry and partly in the weapons production itself. Since, for economic reasons, the labor of the inmates should be preserved, a health care was established (1). At the time of the dissolution of the Mittelbau-Dora concentration camp ten barracks existed in the main camp, in which about 1,300 patients were housed (2, 3). According to the historical documents, “Häftlingskrankenbau Dora” (4) was the most common official name of this place. It could be translated as the Dora prisoners' infirmary or hospital. Since infirmary corresponds more with the conditions than hospital, we decided on the first term.

The scholarly works on the subject of medicine under National Socialism is in the meantime very extensive and difficult to survey. Within the history of medicine, this is the topic that has been most extensively researched in recent years. With regard to concentration camps, the focus of previous medical history research has been predominantly on inhumane medical experiments. On the other hand, the daily medical routine in the concentration camps has hardly been investigated (5). The aim of this article is to analyze the everyday life of patients with tuberculosis in the Mittelbau-Dora concentration camp by means of historical medical records, documents from SS doctors, testimonies, survivor memoirs and contemporary textbooks. The causes, the structure, the medical-nursing treatment and the consequences are examined more closely. As part of a doctoral dissertation, the topic was investigated with regard to pneumonia, tuberculosis and phlegmon (6) and thus provided the basis for this article.

## Materials and Methods

If one looks at the surviving sources of health care in the Mittelbau-Dora concentration camp, it becomes clear that almost exclusively inpatient treatment was documented, which is why the focus of this work is on inpatient treatment. However, it should not be forgotten that a large part of the sick prisoners were not admitted to the prisoners' infirmary. Others were only treated on an outpatient basis for which no documentation was handed down.

In order to investigate the topic, a source-critical-hermeneutic analysis of written sources and transcribed oral history collections from various, partly international, archives was carried out (7). These sources include medical records, documents of the concentration camp administration, correspondences of SS physicians, contemporary medical textbooks, testimonies in post-war trials and survivor memoirs of former concentration camp inmates. Although numerous documents were destroyed by the perpetrators at the end of the war, in Mittelbau-Dora many medical records have been handed down in comparison to other concentration camps.

**Figure 1:**
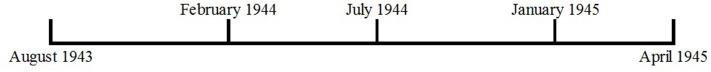
Timeline existence of the Mittelbau-Dora concentration camp and examined months of patients with tuberculosis.

**Figure 2:**
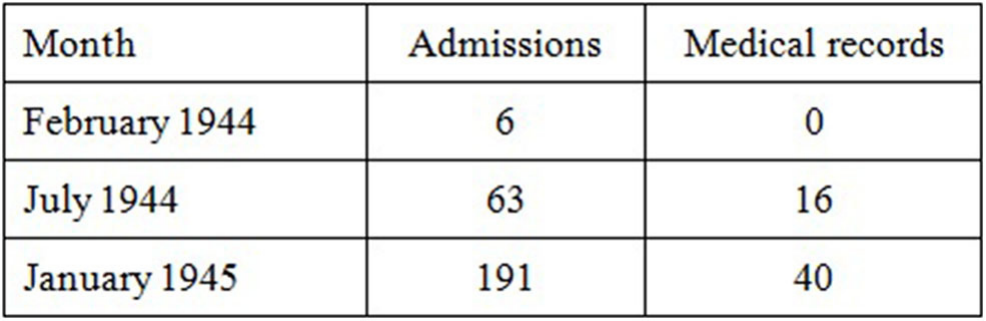
Number of inpatient admission and preserved medical records of patients with tuberculosis in Dora prisoner's infirmary.

After World War Two the historical documents on the Mittelbau-Dora concentration camp were distributed to various archives. The vast majority of medical documents, especially medical records, are stored in the archives of the International Tracing Service (ITS) in Bad Arolsen, Hesse. This was inaccessible to historians until 2007 and a scientific study of medical documents of the Mittelbau-Dora concentration camp is still expected. Part of these documents are the register of inpatient admission of the Dora prisoners' infirmary. Since they are complete, it is possible to reconstruct when which patient was admitted for medical treatment. The medical records also archived there are a special focus of this work. They documented the patient's history, the physical examination, the diagnostic and therapeutic steps in the further course. Because of the large extent of the individual-related documents (328,341 pages)^1^, which includes medical records, first the subject of investigation was restricted. The tuberculosis of the lungs was chosen to be analyzed because it was the most common inpatient treatment according to monthly reports (8). Scholarly works regard tuberculosis as a classic concentration camp disease which was caused by miserable hygienic conditions, cramped living circumstances and malnutrition (9). Since the history of the concentration camp can be divided into three phases which can be defined as build-up, production and dissolution phase the three representative months of February 1944, July 1944, and January 1945 were examined (Figure 1). The number of source of the tuberculosis medical records varies in all months: in February 1944, no records of six admitted patients have survived; for July 1944 there are 16 medical records of 63 admissions; for January 1945, 40 medical records were kept of 191 treated patients. Thus, about 22% of medical records have been preserved (Figure 2). The names of the patients in the medical records were anonymised to protect the personality rights.

Contemporary German medical textbooks are included in the analysis of diagnostic and therapeutic measures (10). In addition, correspondences of the SS doctors are examined (11). Another genre of sources are the documents and testimonies of the post-war processes, which include the Dora Trial held in Dachau (1947) (12) and the Dora Trial held in Essen (1967–1970) (13), which dealt with the crimes committed in the Mittelbau-Dora concentration camp. Finally, the contemporary documents, mostly produced on behalf of the SS, are contrasted with ego documents of former patients with the limitation that these were usually made retrospectively after the liberation. Unfortunately in the case of tuberculosis, there were only a few survivors of this disease, and thus only a very limited number of such reports have emerged. Furthermore, there are survivor memoirs of the inmate medical staff, which consisted mainly of prisoner-functionaries. Few of these reports have been published. A large number of unpublished reports can be found in the Documentation Department of the Mittelbau-Dora Concentration Camp Memorial in Nordhausen (DMD) or in the World Holocaust Remembrance Center Yad Vashem memorial in Jerusalem.

By reconstructing the everyday medical life from the above-mentioned sources, a multi-perspectival examination occurs, in which the perspective of the medical staff (SS and prisoner-functionaries) and the patients is contributed. In the discussion part of the article, Mittelbau-Dora is compared with the main camps of the other concentration camps. Since the situation in the subcamps was very heterogeneous and no medical care was intended in the extermination camps, these are not included in the discussion.

## Results

### Inspections by SS Hygienists Measures Against the Tuberculosis Epidemic

A change of function began with the looming defeat in the Second World War in the concentration camps, in which the production of war-important goods received an ever higher priority. In the course of this, the health care in all concentration camps was reorganized (14). Since the Mittelbau-Dora concentration camp was in a provisional underground tunnel system between August 1943 and June 1944 (15), miserable conditions prevailed. High-ranked hygienists of the Waffen-SS such as Karl Gross (1907–1967), Erwin Ding-Schuler (1912–1945) and Joachim Mrugowsky (1905–1948) inspected the place because the production of the rocket [called by the propaganda “Vergeltungswaffe 2” (revenge weapons) or “V2”] were at risk due to infectious diseases such as tuberculosis and a high death rate. They reported to the Leitenden Arzt der Konzentrationslager (chief physician of the concentration camps) Enno Lolling (1888–1945) and to higher posts of the SS medical service, such as the Reichsarzt SS und Polizei Ernst-Robert Grawitz (1899–1945) (16). In terms of space and staff, medical care was to be expanded. Seriously ill prisoners should be sorted out in regular health appeals (17). Gross explains the purpose of this selection: The aim is “(…) eine unnötige Belastung des Betriebes mit körperlich mangelhaften Menschenmaterial und eine dadurch bedingte Anhäufung von Arbeitsunfähigen zu vermeiden (…)” (18). (“(…) to avoid an unnecessary burden on the company with physically defective human material and a consequent accumulation of incapacitated work (…)”). If this could not be avoided, the seriously ill inmates should be concentrated in a special camp.

In the spring of 1944, the number of tuberculosis-affected prisoners in the Mittelbau-Dora concentration camp continued to rise (8). In April 1944, the SS hygienist Joachim Mrugowsky states in a report that many of the deceased have suffered from infectious tuberculosis (19). Joachim Mrugowsky writes that tuberculosis represents a real danger to the Mittelbau-Dora concentration camp (19). Since the infection endangered the rocket production, patients with tuberculosis should no longer be employed in the underground production facilities. Mrugowsky demanded the installation of an X-ray apparatus, which was to be delivered as soon as possible and became the central selection tool. In addition, in order to raise the pulmonary status of all inmates, trains of the SS-Röntgensturmbann (SS X-ray unit) should be sent for a serial X-ray examination in the Mittelbau-Dora concentration camp (16, 19). Obtaining an adequate X-ray equipment got a high priority, of which the head physician of the concentration camps, Enno Lolling, personally took care. Furthermore, a microscope was needed for the microbiological examination of tuberculosis, which was supplied from Buchenwald (16, 20). From April 1944, the X-ray apparatus was used (21). In June 1944, the serial X-ray examination of all SS members, soldiers of the Wehrmacht and concentration camp inmates started (22). However, the evaluation of the X-rays should have lasted until October. Radiological correlates of tuberculosis were diagnosed in 167 inmates in the inmate infirmary and in 1,371 inmates outside the inmate infirmary (23). In the summer and autumn of 1944, tuberculosis was still one of the central diseases and causes of death (8). Gross' original idea of setting up a special camp for the seriously ill, like most of the patients with tuberculosis, continued to mature until it was finally implemented in the spring of 1945 by using part of the subcamp Boelcke-Kaserne (Boelcke barracks) (24). Actually, the seriously ill should be completely deported from the Mittelbau-Dora concentration camp. Already between January and March 1944, 3,000 seriously ill people were deported to camps with even worse conditions (25). In March 1945, this practice was taken up again (26).

### Places of Treatment of Tuberculosis

Shortly after the foundation of Mittelbau-Dora, the sick were housed in tents (2). At the end of October 1943, the first wooden barrack (called “Block”) was built (27). Tuberculosis patients were initially not accommodated separately. In July 1944, the “Infektionsabteilung” (Infection Department, Block 39) and the “Schonungsblock” (block for convalescents, Block 23) were used to house tuberculosis patients (28). In October 1944, finally, a separate “Tuberkuloseabteilung” (Tuberculosis Department, Block 39 A) was completed (29). This had three rooms for non-infectious tuberculosis and a large hall for patients with infectious tuberculosis (30).

### Medical Staff in the Tuberculosis Treatment

The SS medical staff directed and monitored the medical care of the prisoners (31, 32), but medical treatment by the SS is not documented in the sources. While SS physicians rarely conducted visits to the prisoner infirmary, they were almost never in the Tuberculosis Department (Block 39 A) (33). Because of the infectiousness, the SS staff avoided this place almost completely. The former inmate nurse Paul-André Lobstein (1923–2012) describes the only visit of the SS camp doctor: “Le Lagerarzt s'y aventura un jour, mais, à la vue des Musulmans, couchés à deux par paillasse et nus sous leurs couvertures, il se retira précipitamment” (34). (“The camp doctor came in one day, but on the visit of the ‘Muselmänner', in pairs on a straw sack and naked under the blanket, he took flight”). Thus, the treatment of the inmates was in the hands of prisoner-functionaries, the international inmate medical staff, which in Mittelbau-Dora consisted almost entirely of political prisoners (35). Supreme instance was the Kapo of the prisoners' infirmary (36). Inmate doctors visited, determined the therapy and decided on the dismissal (33). Nursing activities were carried out by male inmate nurses (32). Finally, it was important for the functioning of the infirmary to have other functional positions, such as pharmacists, laboratory technicians or interpreters (35). Initially an SS medic and an inmate doctor with only a few nurses covered the entire medical supply while the number of inmate medical staff in the following months grew more and more (2, 35). An SS camp physician took over the overall management from October 1943 (27).

The workforce of inmates of the prisoners' infirmary consisted of 55 people, including two nurses and two doctors in the Infection Department (Block 39) treating infectious diseases such as tuberculosis in May 1944. One month later, although the staff in the entire infirmary grew by ten people, one prisoner-physician was reduced in the Infection Department (35). In July 1944 an inmate radiologist was sent to the Dora prisoners' infirmary (37). This person was moved from Buchenwald to Mittelbau-Dora as part of the transfer system for medical professionals, organized by the SS chief physician of the concentration camps Enno Lolling (1888–1945) (38). Finally, at the end of March 1945, a total of 40 inmate doctors and 88 inmate nurses worked in the Dora prisoners' infirmary. In the Tuberculosis Department (Block 39 A) there were nine prisoners in medical functions, including three doctors, one upper nurse and five nurses (35).

While the inmate physicians usually had completed medical studies prior to their arrest (2, 39), nurses' former jobs could range from laymen to skilled workers. Due to the limited source, the origin and training of the staff that treated patients with tuberculosis should be presented here using two examples. The inmate nurse Adolf Lindenbaum (born 1921), for example, worked from early 1945 in the Tuberculosis Department (Block 39 A).

He was actually a gardener. As a jew he was arrested 1939. Since Lindenbaum was already employed in the Auschwitz-Monowitz prisoners' infirmary, he was trained in a 2-month course in Auschwitz concentration camp by prisoner doctors in nursing (40). Another inmate nurse that worked in the Tuberculosis Department (Block 39 A) from October 1944, Paul-André Lobstein, was in the middle of his medicine studies before being arrested for political reasons in France in 1943 (30).

### The Medical-Nursing Treatment of Patients With Tuberculosis

The daily routine in the Tuberculosis Department (Block 39 A) was well-structured. In the morning the sick persons were woken up for their washing. The patients received a coffee substitute and the vital parameters were measured. The inmate physicians visited only part of the tuberculosis patients every day. X-rays were ordered and sputum was taken to the laboratory for examination. For lunch, there was often soup and then a bed rest. In the afternoon, body temperature and heart rate were measured again. For dinner patients received bread and margarine. This was followed by the night rest (30, 41).

In the 1940s, tuberculosis was diagnosed on the basis of typical patient's history and physical examination. It could appear like pneumonia or bronchitis. For any suspicion, an X-ray should be taken to confirm the diagnosis and the sputum should be analyzed with special staining, like Ziehl-Neelsen. If *Mycobacterium tuberculosis* could be detected in sputum, infectious tuberculosis was diagnosed. From the X-ray one could deduce extent and prognosis. The suspicion of a non-infectious tuberculosis, however, could only be made by means of typical radiological changes. Furthermore, non-specific examinations such as blood count and erythrocyte sedimentation rate gave conclusions about the severity or activity (10).

In February 1944, no medical records of patients with tuberculosis were preserved in the Dora prisoners' infirmary. Among the many pneumonias there were undoubtedly misdiagnosed cases of tuberculosis as the autopsies suggest (19). In July 1944, tuberculosis patients were treated in the Infection Department (Block 39). For each patient, a physical examination is documented once a week. Other tuberculosis patients were housed in the “Schonungsblock” (Block 23), where no examinations are documented. This shows the different treatment depending on the department. Almost all inmates with tuberculosis received a chest X-ray, which was usually taken shortly after hospitalization, and the sputum was examined for *Mycobacterium tuberculosis*. An infectious tuberculosis was frequently found. Finally, the erythrocyte sedimentation rate was determined (28, 42). In January 1945, the three diagnostic means chest X-ray, sputum analysis and erythrocyte sedimentation rate were often performed on the day of admission. From early on the infectivity and the severity of tuberculosis should be determined. In addition, physical examinations are documented. There were differences in frequency depending on the department block. While in the Infection Department (Block 39), where non-infectious tuberculosis patients with good prognosis were housed, a medical examination is documented every 4–5 days, in the Tuberculosis Department (Block 39 A), where infectious or severely affected tuberculosis patients were found, physical examinations usually only occurred every seven days (28, 43).

In the survivor memoirs of former inmates there is a description of the X-ray station of the Dora prisoners' infirmary. Since Boris Pahor (born 1913) was suspected of having tuberculosis, his lungs should be X-rayed. He describes how the inmate radiologist worked while a great pile of corpses was burning behind the barrack: “Sein Tun glich also dem Eifer eines Arztes, der in einem aufgegebenen U-Boot die Lungen der Mannschaft untersucht. Ich weiß nicht, was er festgestellt hat, auf jeden Fall war er in fünf Minuten mit mir fertig” (44). (“His work was similar to the enthusiasm of a doctor examining the crew's lungs in a derailed submarine. I do not know what he found out, but in any case, he was done with me in five minutes”). The former inmate nurse Paul-André Lobstein worked in the Tuberculosis Department (Block 39 A) from October 1944. He reports how patients were selected by the diagnostics: “Dépistés par la radioscopie, les malades nous arrivaient de la baraque des infectieux avec le diagnostic de tuberculose, et suivant que leurs crachats étaient négatifs ou positifs, ils séjournaient dans l'une des petites salles ou en moyenne six semaines dans la grande. De là, ils voyaient disparaître, un à un leurs compagnons, enlevés par la mort” (45). (“Sorted by X-ray, the sick came to the infectious block with the diagnosis of tuberculosis. Depending on whether their sputum was negative or positive, they were housed in one of the small rooms or in the large hall for an average of 6 weeks. Here they saw one after the other of their fellow inmates disappear, snatched away by death”).

In the 1940s different treatments of tuberculosis existed. For some patients, a hygienic dietary treatment that included rest, fresh air and plenty of food was recommended. If the patient was stable, it was recommended to take a rest cure in the open air. In addition, as part of the symptomatic therapy fever, cough, pulmonary hemorrhages, circulatory weakness and other symptoms were treated. Furthermore, there was a non-specific therapy. In order to stimulate the immune system, tuberculin or sunlight exposure or artificial exposure to sunlamp, a device for generating UV radiation, or X-rays were used. Another unspecific therapy was the collapse therapy. By compressing lung areas, the pulmonary tuberculosis focus should be cured. Depending on the exact location and severity of the tuberculosis disease, there were four different compression methods, including artificial pneumothorax, phrenicus exhairese, thoracoplasty, or sealing. A specific therapy against *Mycobacterium tuberculosis* was unknown at the time (10).

In July 1944, tuberculosis was primarily treated symptomatically like with chest compresses or the antipyretic Pyramidon. In the Infection Department (Block 39), all patients with tuberculosis received daily antipyretic measures, cough suppressants whose ingredients are not specified, and vitamins. There, the patients were given an injection with calcium gluconate, which, according to evaluation in the textbooks, was used for the prophylaxis and treatment of pulmonary hemorrhages (10). In patients of the “Schonungsblock” (Block 23), these agents were used very rarely. Thus, only inmates who were admitted to the Infection Department (Block 39) received all funds available in the Dora prisoners' infirmary (28, 42).

In January 1945, inmates with tuberculosis were treated in the Infection Department (Block 39) or the Tuberculosis Department (Block 39 A). Lightly sick and non-infectious patients came to the Infection Department (Block 39) where they were daily given cough medicine, vitamins and calcium. Sunlamps and intravenous injections of calcium chlorate or gluconate were applied in the distance of 5 days. The installation of an artificial pneumothorax was carried out in a few inmates. In contrast, there was almost no therapy in the Tuberculosis Department (Block 39 A), where the seriously ill and infectious tuberculosis patients were admitted. Whoever came there was therefore significantly worse supplied (28, 43).

There are only a few reports available from the perspective of tuberculosis patients in the Dora prisoners' infirmary. This obvious gap can be explained by the high death rate. In addition, many tuberculosis patients were selected for extermination transports, which they usually did not survive (30, 46, 47). One of the few survivors is Viktor Bender (born 1924), who was in the Infection Department (Block 39) at the end of 1944. He remembers that usually three inmates had to lie in one bed, the food had an inferior quality and the severely ill patients vegetated lethargically (48). The former inmate André Mouton (1924–2017), who observed the tuberculosis patients, reports about the treatment of sunlight in the open air in the summer of 1944: “Sie waren vollkommen nackt. Sie setzten sich auf die Steine, wo sie blieben, ohne sich bewegen zu können. Denjenigen, denen es gelang sich aufrecht zu halten, vermittelten einen furchtbaren Eindruck” (49). (“They were completely naked. They sat down on the stones, where they stayed without being able to move. Those who managed to keep up made a terrible impression”). Furthermore, there are reports from the perspective of the inmate medical staff. The former inmate nurse Paul-André Lobstein explains that the Tuberculosis Department (Block 39 A) was known in the entire concentration camp because of its poor conditions, so the staff was very limited in their possibilities: “Mais la gravité extrême des cas et la pénurie en médicaments rendirent vite, surtout à la fin, à peu près illusoire pour le personnel médical tout rôle autre que celui d'une assistance morale” (45). (“But the severity of the cases and the paucity of medication quickly, especially toward the end, made any role of medical staff illusory except for moral support”). Although the lack of everything was reality in health care, the care of inmate medical staff played an important role, as the former inmate Max Dutillieux (1923–2003) describes: “Un jeune médecin tchèque qui parle très bien le français vient me rendre visite tous les soirs. Il m'apporte un grand bol de lait. Ceci m'a sans doute sauvé: cinq minutes de présence amicale, des paroles réconfortantes, un sourire et un bol de lait” (50). (“A young Czech doctor, who speaks very good French, comes to visit me every evening. He brings me a big bowl of milk. That has without doubt saved me: five minutes presence of a friend, comforting words, a smile and a bowl of milk”).

In summary, a tendency toward professionalization of the treatment can be seen over the 3 months, but this was undermined by the increasing lack of material. Over the entire period, however, the limited resources were only available to the sick who were classified as curable. X-rays and sputum examinations were therefore the central selection instruments in July 1944 and January 1945.

### The Consequences of the Treatment of Tuberculosis

Compared to other diseases, tuberculosis required a long stay in the Dora prisoners' infirmary. For February 1944 no files are handed down. In July 1944, patients were hospitalized from 1 to 5 months. In January 1945, the duration was between 2 and 5 weeks. The longest stay of a patient found in this investigation was 248 days (51). Because of the lack of medication there were sometimes long disease courses. In most cases, rest was the only therapy option. The patients were weakened and powerless and often had an infaust prognosis. They got a special status because of the infectiousness (28, 42, 43).

**Figure 3:**
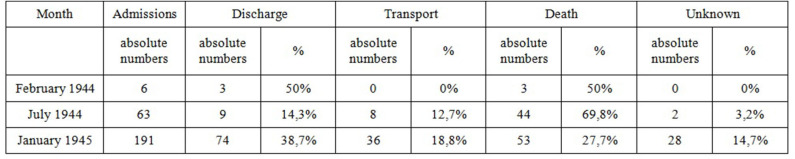
Outcome of treatment of patients with tuberculosis in Dora prisoner's infirmary.

Looking at the register of inpatient admission to the Dora prisoners' infirmary, the consequences of the treatment can be understood for the majority of patients with tuberculosis (Figure 3). For some, however, the whereabouts are undocumented (3.2% in July 1944, 14.7% in January 1945). Of the inmates with tuberculosis admitted in July 1944, only 14.3% were discharged directly. 69.8% died during their stay in the inmate infirmary during the following weeks. After a long stay eight prisoners (12.7%) were selected for an extermination transport. In January 1945, 38.7% of patients with tuberculosis returned to their work squad. During this time, long-term sick were released early due to overcrowding, which did not mean that they were cured (52). 27.7% died and 18.8% were selected for an extermination transport (28, 43). Overall, it becomes clear that the diagnosis tuberculosis had a very poor cure rate.

There were already extermination transports for the long-term sick, each with 1,000 inmates, on 6 January 1944, on 6 February 1944 (both to the Lublin-Majdanek concentration camp) and on 27 March 1944 (to the Bergen-Belsen concentration camp) (25). These camps served as a collective camp for the seriously ill within the concentration camps (53). From the medical records, the selection for an extermination transport can be noticed by the marking with a T. Many detained prisoners died as a result of desperate conditions, neglect or killing (54). Especially patients with tuberculosis were to be removed with these transports from Mittelbau-Dora Concentration Camp. The SS camp doctor Karl Kahr (1914–2007) later reported as a witness on the transport in March 1944: “Since it was a known fact right from the start that it was a transport of TBC patients with the added remark, “open TBC” it was known right from the start that it was a question of hopelessly ill people because the person who has open TBC evidently is very seriously ill” (55). In part, they were also referred to as tuberculosis transports (2). From March 1945, seriously ill patients were again deported to the subcamp Boelcke-Kaserne, where they mostly died of permanent neglect. A part was brought in a transport of 2,252 inmates from there on March 8, 1945 in the concentration camp Bergen-Belsen (28, 30, 43, 46, 47). The conditions in the subcamp Boelcke-Kaserne Nordhausen are described by the former prisoner nurse Siegfried Halbreich (1909–2008): “The ground floor was covered with straw, and hundreds of men laid on the ground. The hangar smelled from rotting bodies full of respiratory ailments, typhus, and a host of other diseases” (56). There was no medical care there (47). Victor Bender, who suffered from tuberculosis, also recalls: “Wer dorthin hereinkommt, kommt nicht mehr heraus. Ich war ziemlich, ein paar Wochen müsste ich dort gewesen sein, zwei, drei, vier Wochen, ich weiß nicht. Wir haben 'rum vegetiert dort auf dem Fußboden” (48). (“Those who come in there cannot get out. I was pretty sure, I must have been there for a couple of weeks, two, three, four weeks, I do not know. We've vegetated around there on the floor”).

Overall, at the end of the war, so many patients with tuberculosis were deported from the Dora prisoners' infirmary that the Tuberculosis Department (Block 39 A), compared to other departments, was nearly empty. Inmate nurse Paul-André Lobstein reports: “Il ne resta de tout le block 39 A, soudain désert, qu'une trentaine de mourants, qui furent libérés par les Alliés, et une interminabile liste de morts” (57). (“From the entire block 39 A, suddenly deserted, remained no more than about 30 dying, who were liberated by the Allies, and an endless list of the dead”).

## Discussion

Tuberculosis has always been given great importance in National Socialist health policy. Patients were stigmatized by the Nazis as inferior and a genetic component assumed. Thus, they also had to endure coercive measures in civilian life (for example restrictions on marriage, compulsory hospitalization), which often led to social isolation (9, 58, 59). Here it can be shown that tuberculosis in the Mittelbau-Dora concentration camp was identified as one of the central threats to armaments industry by SS hygienists. Inspections by SS hygienists are not yet documented for other concentration camps. The aim of the inspections, however, was not to create more humane conditions, but to increase prisoner forced labor. In order to achieve the early diagnosis of diseases for the “Wiederherstellung” (60) (“restoration”) of the workforce.

Their reports and proposed countermeasures led to structural and staff expansion of health care in Mittelbau-Dora concentration camp. To identify tuberculosis the prisoners' infirmary was extended by an X-ray apparatus and a microscope. Serial X-ray examinations are already documented for Mittelbau-Dora (61) and described for other concentration camps such as Buchenwald (62), Dachau (63), and Sachsenhausen (64). The research on Mittelbau-Dora has already dealt with the construction of an alternative camp for inmates labeled “unfit for work” (65).

As it can be shown here, patients with tuberculosis were housed in Mittelbau-Dora in the Infection Department (Block 39), the Tuberculosis Department (Block 39A) and the “Schonungsblock” (Block 23). A similar structure with a separate department for infectious and tuberculosis patients within the inmate infirmary was also available in Dachau (63) and Sachsenhausen (9). For Mittelbau-Dora (61, 66) and other concentration camps the existence of a X-ray apparatus [Auschwitz-Monowitz (67), Auschwitz-Stammlager (68), Buchenwald (69), Dachau (63), Flossenbürg (70), Natzweiler-Struthof (71), Neuengamme (72), Ravensbrück (73) and Sachsenhausen (74)] and laboratories [Auschwitz-Stammlager (68), Buchenwald (69), Natzweiler-Struthof (71), Ravensbrück (73) and Sachsenhausen (74)] are already verified. Here, however, it becomes clear for the first time which high priority the setting up of X-ray apparatus and laboratory had for the diagnosis of tuberculosis in Mittelbau-Dora.

The health care in Mittelbau-Dora was supervised by the SS, but the actual treatment was carried out exclusively by functional prisoners. The previous research on other concentration camps (63, 72, 74, 75) comes to a similar result, whereas the previous research on Mittelbau-Dora (61, 66) refers to frequent (and not exclusive) treatment by prisoners. In the present article it can be shown that over the course of time more and more staff in the Dora prisoners' infirmary was employed. In part, this staff came through the targeted transfer of medical professionals from other concentration camps to Mittelbau-Dora. Compared to the other departments of the Dora prisoners' infirmary the low number of staff in the infectious diseases shows that curing did not have any importance. In contrast, the high priority given to diagnostics is evident from the fact that an inmate radiologist was sent to the Dora prisoners' infirmary from another concentration camp.

Previous studies reveal that only from 1942 approved doctors or medical students were allowed to work in the health care (72, 76). On the other hand, SS medical staff was present only in small numbers over the entire period. This SS staff shortage is also described for other concentration camps (59, 75). Unfortunately, a comparison of staff development in the Tuberculosis Department cannot be carried out for the lack of comparable studies.

In this study, for the first time in the concentration camp research, a disease is analyzed on the basis of the systematic evaluation of historical medical records. In February 1944, no medical records could be found, mainly because the limited options of diagnosis and the lack of qualified staff made the disease hardly distinguishable from other lung diseases. By analyzing the medical records of June 1944 and January 1945 it becomes clear that X-ray and laboratory diagnostics were used primarily as selection tools to decide among treatment or neglect. Thus, infectious patients and severe illnesses were identified. While diagnostics had a high status, therapeutic options were very limited. Most cases of tuberculosis were treated symptomatically with antipyretic and antitussive remedies. To strengthen the immune system, vitamins, sunlight and sunlamps were used. On this point, the previous research (77) on the Mittelbau-Dora concentration camp can be refuted, where it is determined that generally no drugs were used to treat tuberculosis.

Here can be shown that there were different therapeutic approaches depending on the accommodation in a department in the Dora prisoners' infirmary. While the Tuberculosis Department (Block 39 A) was used as an isolation and neglect site for patients with poor prognosis, tuberculosis patients with better healing prospects in the Infection Department (Block 39) had significantly more resources available. It can also be shown that there was a graduated system of transfer depending on the prospects of recovery within the departments of the Dora prisoners' infirmary. When the disease state changed, the patients could be transferred from one department to another. It can be agreed to former research that the Tuberculosis Department (Block 39 A) thus played a special role (78). In the existing research, the term “Sterbezone” (“death zone”) has been introduced for these areas in the concentration camp (61). The treatment of tuberculosis has so far only been described for a few concentration camps. In Ravensbrück no medication was used and in the Tuberculosis Department there was permanent overfilling. Tuberculosis patients only got an extra mucus suppository diet in good prognosis (79). In Sachsenhausen, on the other hand, a minimal supply was described with the focus on bed rest and symptomatic therapy, similar to Mittelbau-Dora (9). In January 1945 in some cases collapse treatment by means of an artificial pneumothorax was used. That was also stated for Dachau (63) and Sachsenhausen (9, 74). Also for the Dachau concentration camp, a selection of curable and incurable forms of the disease by chest X-rays is already pointed out (63). Curable cases were treated in the prisoners' infirmary, incurable patients were housed in the “Invalidenblock” and often killed with injections.

From the sources examined here, in comparison to other concentration camps (for example Auschwitz, Buchenwald, Dachau, Neuengamme, Mauthausen, Majdanek and Sachsenhausen) (59), no experiments can be found on tuberculosis patients in Mittelbau-Dora.

While the previous research (78) on Mittelbau-Dora represents long stays in the prisoners' infirmary as an isolated case, it is shown here that this was a frequent phenomenon, especially for patients with tuberculosis. The duration of inpatient treatment in other concentration camps has hardly been investigated so far. In some prisoner' infirmaries, the treatment was allowed to last a maximum of 2–4 weeks [Auschwitz-Stammlager (80) and Auschwitz-Monowitz (67)] or a maximum of 3 months [Dachau (63) and Mauthausen (81)]. The sick were often classified as “invalid,” transported to central camps for seriously ill prisoners or killed. Patients with tuberculosis were often long-term sick prisoners who were classified by the SS as „arbeitsunfähig“ (“incapacitated for work”). These were killed in the same concentration camp, transported to a camp for seriously ill prisoners or killed elsewhere. Between 1941 and 1942 the “Aktion 14f13” occurred, in which patients from ten concentration camps (Auschwitz, Buchenwald, Dachau, Flossenbürg, Groß-Rosen, Mauthausen, Neuengamme, Niederhagen, Ravensbrück and Sachsenhausen) were deported to Bernburg, Hartheim or Sonnenstein for killing (76). From the spring of 1943, this “Aktion 14f13” was limited by Heinrich Himmler (1900–1945) to mentally ill prisoners, since the labor of the others should be exploited till the end and if necessary also in the hospital bed (63, 68, 73, 74, 76). For this reason this “Aktion 14f13” did not apply in the Mittelbau-Dora concentration camp. In addition, in nearly all concentration camps from the late summer of 1941 there were regular killings of patients by injections, shootings or gas (76). The systematic killing of the sick cannot be determined in the Dora prisoners' infirmary.

Starting from the summer of 1940, the extermination transports (61, 66) were another method used by the SS to deport severely ill prisoners. The patients were transported to special concentration camps (1940–1942 Dachau, December 1943–March 1944 Lublin-Majdanek, March 1944–1945 Bergen-Belsen) with even worse conditions, which few survived, so that these transports amounted to a death sentence. In these camps the sick were killed or died of neglect (76). In this work it is shown that especially tuberculosis patients from the Dora prisoners' infirmary were selected for these extermination transports (January-March 1944 and March 1945). These transports have already been described in other works on Mittelbau-Dora (61, 66, 76), which were also carried out in other concentration camps (9, 72). Finally, it can be stated that from March 1945, patients with tuberculosis were collected in the subcamp Boelcke-Kaserne. Scholar work already found out that it served as a death camp for the sick and the weak of the entire Mittelbau-Dora concentration camp complex (61, 66, 76).

This study shows that research on the history of concentration camps remains important. The historical example demonstrates that nutrition, hygiene and health care are universal rights to which all people, whether imprisoned or free, should have free access. Conditions in prison camps should be critically examined on a regular basis.

## Data Availability Statement

The datasets generated for this study are available on request to the corresponding author.

## Ethics Statement

Ethical review and approval was not required for the study on human participants in accordance with the local legislation and institutional requirements. Written informed consent from the participants was not required to participate in this study in accordance with the national legislation and the institutional requirements.

## Author Contributions

PK and FS contributed to the design and implementation of the research, to the analysis of the results and to the writing of the manuscript. All authors contributed to the article and approved the submitted version.

## Conflict of Interest

The authors declare that the research was conducted in the absence of any commercial or financial relationships that could be construed as a potential conflict of interest.

## Footnotes

^1^https://arolsen-archives.org/suchen-erkunden/sucheonline-archiv/gesamtinventar/ (accessed August 11, 2020).
